# DOA Estimation Method Based on Improved Deep Convolutional Neural Network

**DOI:** 10.3390/s22041305

**Published:** 2022-02-09

**Authors:** Fangzheng Zhao, Guoping Hu, Chenghong Zhan, Yule Zhang

**Affiliations:** 1 Graduate School, Air Force Engineering University, Xi’an 710043, China; zhaofz1020@163.com (F.Z.); chenghong_zhan@163.com (C.Z.); yule_zhang0921@163.com (Y.Z.); 2 Air and Missile Defense College, Air Force Engineering University, Xi’an 710043, China

**Keywords:** DOA estimation, deep convolutional neural network, covariance matrix, the upper triangular matrix

## Abstract

For the multi-target DOA estimation problem of uniform linear arrays, this paper proposes a DOA estimation method based on the deep convolution neural network. The algorithm adopts the deep convolutional neural network, and the DOA estimation problem of the array signal is transformed into the inverse mapping problem of the array output covariance matrix to a binary sequence in which “1” indicates that there is a target incident in the corresponding angular direction at that position. The upper triangular array of the discrete covariance matrix is used as the data input to realize the DOA estimation of multiple sources. The simulation results show that the DOA estimation accuracy of the proposed algorithm is significantly better than that of the typical super-resolution estimation algorithm under the conditions of low SNR and small snapshot. Under the conditions of high SNR and large snapshot, the estimation accuracy of the proposed algorithm is basically the same as those of the MUSIC algorithm, ESPRIT algorithm, and ML algorithm, which are better than that of the deep fully connected neural network. The analysis of the simulation results shows that the algorithm is effective, and the time and space complexity can be further reduced by replacing the square array with the upper triangular array as the input.

## 1. Introduction

Array signal processing, also known as spatial domain signal processing, is an important branch of the signal processing field, widely used in radar signals, underwater sonar, wireless communication, radio astronomy, and other fields [1]. Mainly, to process the signals received by the array, enhance the useful signals needed, suppress useless interference and noise, and obtain important parameters, the estimation of the direction of arrival (DOA) is one of the important research contents of array signal processing [2]. DOA estimation, also named spatial spectrum estimation, estimates the direction angle of the spatial signal reaching the array reference element by processing the received signal of the array. The traditional DOA estimation methods are mainly based on beamforming [3] and ‘null pattern’ guidance techniques [4], and the typical representatives are the methods of delayed-add and Capon minimum variance [5,6]. In order to maximize the amplification of useful signals and suppress interference signals, this type of method searches for the peak output power and aligns the main lobe with the incident direction of useful signals under the premise of a known array flow pattern. It is only applicable to the case where there is only one signal in the target airspace. In the late 1970s to early 1980s, the development of the proper channeling subspace algorithms made DOA estimation leap to the super-resolution level. The characteristic subspace algorithms are divided into two types, noise subspace algorithms represented by the MUSIC algorithm, and signal subspace algorithms represented by the ESPRIT algorithm. The MUSIC algorithm [7], proposed by Schmidt in 1979, uses the orthogonality of the signal subspace and noise subspace to construct a spatial spectral function and estimate signal parameters through spectral peak search. The ESPRIT algorithm [8], proposed by Roy et al. in 1989, is based on the rotation invariance of the subspace that does not need spectral peak search, and its computing efficiency is greatly improved. In 1988, maximum likelihood estimation theory was applied to DOA estimation [9]. This method still has a good performance under the condition of low SNR and snapshot deficiency. As the maximum likelihood DOA estimation function is nonlinear, multidimensional search is indispensable for solving the optimal solution, which generates a large number of operations; alternating projection [10] is usually used to simplify the optimal solution process. The subspace fitting algorithm [11] is similar to the maximum likelihood algorithm; the difference is, the former is used to fit the signal subspace [12] and noise subspace [13], and the latter is used to fit the received data and actual signal. Both algorithms require multidimensional search; therefore, the solving process of the maximum likelihood algorithm can be directly applied to the subspace fitting algorithm [11].

In recent years, the continuous development of deep learning theory and technology has provided new ideas and strategies for DOA estimation [14]. Deep learning-based DOA estimation methods can be divided into two main research branches: the first is a supervised algorithm that learns the projection relationship between the inputting feature and DOA. For example, in 2015, a single-layer neural network model was designed by Xiao [15] to implement DOA estimation, and the deep learning method was used to solve the DOA estimation problem for the first time. In addition, in 2018, CNN was introduced into the study of the DOA estimation problem for the first time by Chakrabarty and Habets [16], which improved the estimation accuracy and robustness of the algorithm. In addition, neural networks dealing with higher-order fractional linear systems such as GMDH neural networks [17] also provide new ideas for supervised DOA-based estimation methods. The second is an unsupervised algorithm based on feature enhancement. For example, in 2020, Xiang proposed an algorithm that improved the estimation accuracy by phase enhancement [18]. Although the existing methods about deep learning have a preferable estimation precision, due to the complex model and overmuch training parameters, time complexity and space complexity are unsatisfactory. Based on this, a DOA estimation method by deep convolutional neural networks (DCNN) is proposed. The upper triangular matrix of the covariance matrix of the discretized received signals is used as the input, which can effectively reduce the number of parameters generated by the full connected layer, and DOA estimation can be achieved at the cost of lower time and space complexity. In addition, the mapping between input and DOA can be obtained by network training.

## 2. Signal Model

Suppose there is an ideal uniform linear array (ULA) with L array elements whose element spacing is less than half a wavelength. M farfield narrowband signals are incident to the array at angles of $\theta_{1},\theta_{2}\ldots \theta_{k}$, space signal sources are fixed, and the center frequency of all signals is the same and known. Under these conditions, the output signal vector of the ULA with the number of snapshots t is as follows:

(1) $$X\left(t\right)={\left[x_{1}\left(t\right),x_{2}\left(t\right)\ldots x_{L}\left(t\right)\right]}^{T}=\sum_{m=1}^{M}a\left(\theta_{m}\right)s_{m}\left(t\right)+n\left(t\right)=A\left(\theta \right)s\left(t\right)+n\left(t\right),$$

where $x_{i}\left(i=1,\;2\ldots L\right)$ is the output of the i’th array element; $\theta_{m}$ is the DOA of the m’th incident signal; m=1,2,…,M; $\theta ={\left[\theta_{1},\theta_{2},\ldots ,\theta_{M}\right]}^{T}$ is the DOA vector; $s\left(t\right)={\left[s_{1}\left(t\right),s_{2}\left(t\right),\ldots ,s_{M}\left(t\right)\right]}^{T}$ is the signal vector; $n\left(t\right)={\left[n_{1}\left(t\right),n_{2}\left(t\right),\ldots ,n_{L}\left(t\right)\right]}^{T}$ is the array additive noise vector; and $a\left(\theta_{m}\right)$ denotes the steering vector of the m’th signal and is expressed below:

(2) $$a\left(\theta_{m}\right)={\left[1,e^{\frac{j2\pi d\sin\theta_{m}}{\lambda_{0}}},\ldots ,e^{\frac{j2\pi \left(L-1\right)d\sin\theta_{m}}{\lambda_{0}}}\right]}^{T},$$

(3) $$A\left(\theta \right)={\left[a\left(\theta_{1}\right),a\left(\theta_{2}\right),\ldots ,a\left(\theta_{M}\right)\right]}^{T},$$

where $\lambda_{0}$ is wavelength of incident signals, d is the element spacing and is not more than $0.5\;\lambda_{0}$ (that is $d\leq 0.5\;\lambda_{0}$), and the covariance matrix $R_{xx}$ of the output matrix X(t) is as follows:

(4) $$R_{xx}=E\left\{X\left(t\right)X^{H}\left(t\right)\right\}=A\left(\theta \right)R_{ss}A^{H}\left(\theta \right)+\sigma^{2}I_{L},$$

where $R_{ss}=E\left\{s\left(t\right)s^{H}\left(t\right)\right\}$ is the signal covariance matrix, $\sigma^{2}$ is an unknown noise power, and $I_{L}$ is the identity matrix of L×L.

The problem of DOA estimation is to obtain the incident angles of the sources aided by the array output signal vector and geometry. Generally, the number of sources is finite, and the signal arrival directions are sparsely distributed in space [19]. Hence, the spatial domain [−60°, 60°] can be divided into N sets of discrete angles $\tilde{\theta }={\left[{\tilde{\theta }}_{1},{\tilde{\theta }}_{2},\ldots ,{\tilde{\theta }}_{N}\right]}^{T}$ with equal spacing:

(5) $$\tilde{A}={\left[a\left({\tilde{\theta }}_{1}\right),a\left({\tilde{\theta }}_{2}\right),\ldots ,a\left({\tilde{\theta }}_{N}\right)\right]}^{T},$$

(6) $$\tilde{s}\left(t\right)={\left[{\tilde{s}}_{1}\left(t\right),{\tilde{s}}_{2}\left(t\right),\ldots ,{\tilde{s}}_{N}\left(t\right)\right]}^{T},$$

(7) $$\tilde{X}\left(t\right)=\tilde{A}diag\left(r_{1},r_{2},\ldots ,r_{N}\right)\tilde{s}\left(t\right)+n\left(t\right),$$

When a signal arrives in a certain direction in space, its position number in the discrete set $\tilde{\theta }$ is $r_{i}=1$; otherwise, $r_{i}=0$. Therefore, the DOA estimation problem is converted into the inverse mapping of the array output covariance matrix to the position ordinal of the corresponding nonzero element in the discrete set. In this paper, the DCNN model is designed to solve the DOA estimation problem, in which the array output covariance matrix calculated by the array output signal vector obtained by Equation (7) is the input of the DCNN model, which is:

(8) $${\tilde{R}}_{xx}=E\left\{\tilde{X}\left(t\right){\tilde{X}}^{H}\left(t\right)\right\},$$

As the covariance matrix has symmetry, the upper triangular array (or lower triangular array) of the covariance matrix is selected as the input of the convolutional neural network and the set of discrete angle numbers ${\left\{r\right\}}_{i=1}^{N}$ as the output of the convolutional neural network in the training phase to reduce the input data size and the operation volume. In the test phase, the nonzero or larger M values of the set of discrete angle numbers ${\left\{r^{\prime }\right\}}_{i=1}^{N}$ are the angle estimates with signal arrival, i.e., ${\left\{{\tilde{\theta }}_{i}^{\prime }\right\}}_{i=1}^{M}$.

## 3. Deep Convolutional Neural Network Model

**Figure 1:**
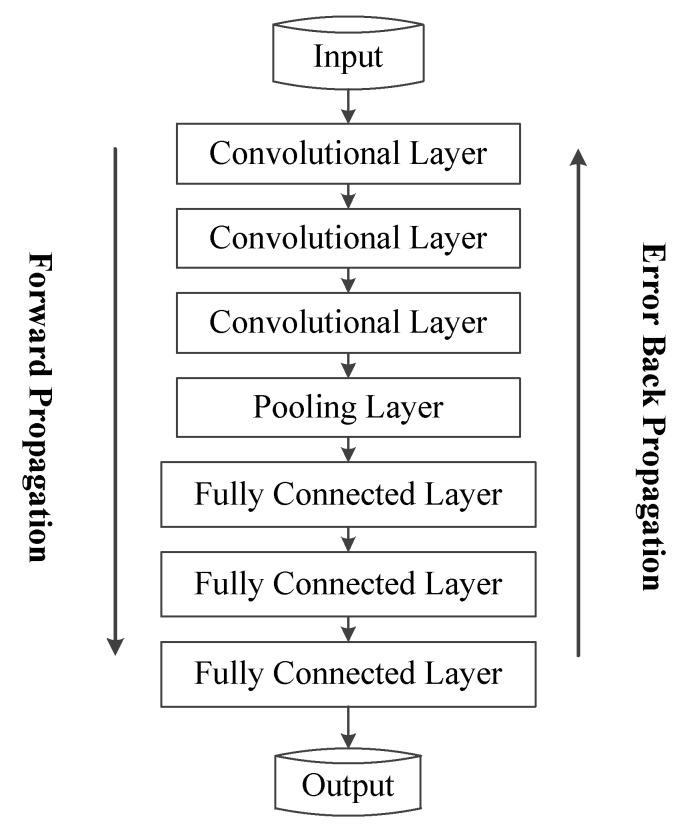
DCNN model.

The convolutional neural network (CNN) is a common deep learning algorithm, which is a feedforward neural network that can reduce the number of parameters to a large extent by local connectivity and weight sharing [20]. A typical CNN model usually consists of several convolutional and pooling layers connected alternately, ending with a fully connected layer [21]. The convolutional layer extracts the features, the pooling layer samples the features, and the fully connected layer connects all the extracted features and finally solves the corresponding problem by a classifier or regressor. Generally, one convolutional layer plus one pooling layer is a feature extraction process, but the pooling layer is not necessary and not always connected after each convolutional layer; it can be designed and selected according to the input data characteristics. In this paper, the DCNN model is as follows in Figure 1.

Deep convolutional neural networks have significant advantages in extracting spatial features, and DCNNs require fewer parameters than fully connected neural networks. The convolutional neural network model contains three convolutional layers, connects a pooling layer, and finally connects three fully connected layers. After forward propagation, the backward error propagation is performed to correct the parameters, iterating in this manner, and the network training is completed when the training error is less than the threshold or the iterations are completed.

### 3.1. Forward Propagation Process

**Figure 2:**
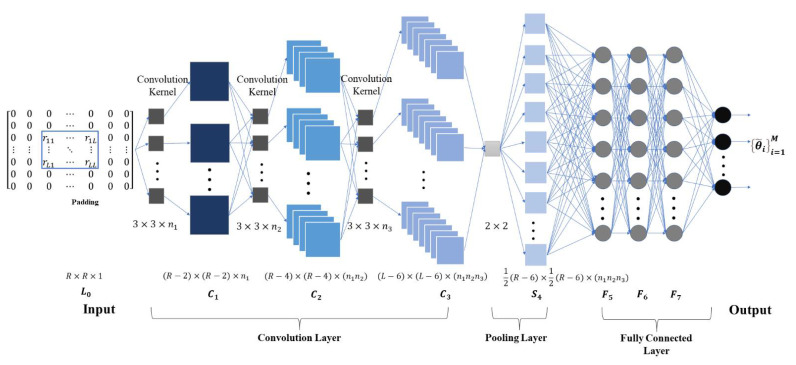
The specific CNN structure.

The specific CNN structure in the forward propagation process is shown in Figure 2.

Three convolutional layers are used for feature extraction, and the sizes of the convolutional kernels are all 3×3, with the numbers of $n_{1}$, $n_{2}$, and $n_{3}$. The role of the pooling layer is to reduce the dimension and prevent overfitting, and as the number of array elements is often not too large, only one pooling layer is added at the end of the convolutional network structure design, with a template of 2×2.

**Figure 3:**
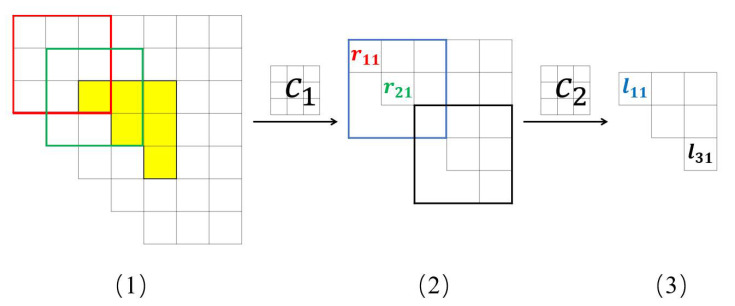
Two convolution processes of the upper triangular array. (**1**), (**2**) and (**3**) in denote the form of the data before and after the convolution operation, respectively.

Before the convolution operation, the input data are first padded in order to preserve the data boundary features. As the convolution kernel size is 3×3, two rows (columns) are filled at each boundary of the input data to preserve the boundary features of the input data. In the convolution layer, sliding convolution calculations are completed according to the convolution kernel size and step size. The convolution kernel is corrected by error backpropagation in the backpropagation stage. The input of the neural network is the upper triangular array of the covariance matrix, and a 3×3 covariance matrix with a step size of 1 is used as an example to perform two convolution processes, as shown in Figure 3.

(1), (2) and (3) in Figure 3 denote the form of the data before and after the convolution operation, respectively. The yellow part of (1) denotes the upper triangular array of the covariance matrix of the neural network input expressed above, and its white part denotes the padding part. The red and green matrices in (1) are convolved with $c_{1}$ respectively, and the results obtained correspond to $r_{11}$ and $r_{21}$ in (2). Similarly, the blue and black matrices in (2) are convolved with $c_{2}$ and the results obtained correspond to $l_{11}$ and $l_{31}$ in (3).

After the convolution operation, bias is added to the convolution result and then input to the activation function to increase the nonlinearity of the system. Currently, most neural networks use the ReLU function as the activation function, but when the data input has negative numbers, the activated result will fall into the hard saturation zone and lose the original feature expression, and the weights will not be able to update in the iterative training, making the relevant neurons lose their functions. Therefore, this paper adopts the Leaky ReLU function as the activation function, and its expression is

(9) $$f\left(x\right)=\{\begin{array}{ll} x & x\geq 0\\ ax & x<0 \end{array},$$

**Figure 4:**
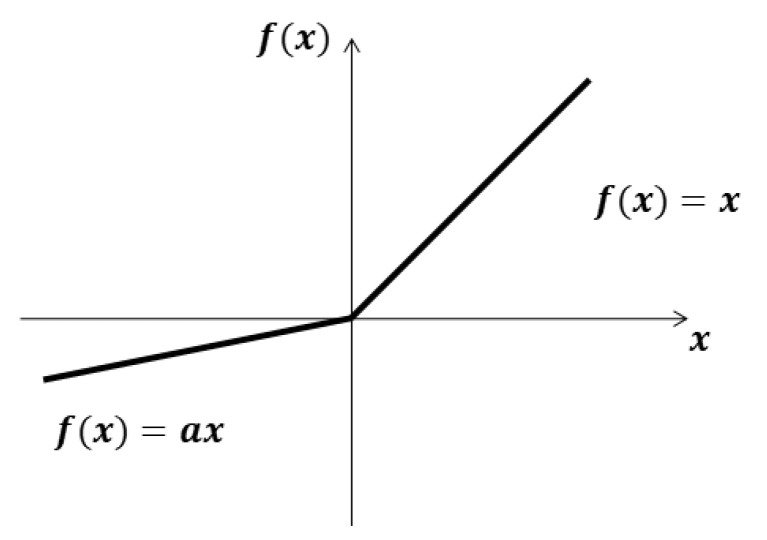
The Leaky ReLU function.

The figure of the Leaky ReLU function is shown in Figure 4.

As can be seen from the Figure 4, when the input value is positive, the result is itself by the activation function output, and when the input value is negative, the Leaky ReLU function assigns a smaller slope. In Equation (9), a is a fixed parameter less than 1 and greater than 0.

In the convolutional neural network structure, the pooling layer is connected after the convolutional layer. The purpose of the pooling layer is to: (1) reduce the number of features and data redundancy; (2) improve data scale invariance and rotation invariance; (3) prevent overfitting. The pooling layer is generally divided into maximum pooling and average pooling with a size of 2×2. The pooling layer does not contain parameters and divides the data into 2×2 subregions starting from the top left of the input data, with no overlap or omission between subregions. Maximum pooling means that the maximum value is retained by internal selection, and average pooling means that the average value of each subregion is retained by calculation. In this paper, maximum pooling is used in the model. As the input of the convolutional layer is the upper triangular array, the input of the pooling layer is also the upper triangular array, and when there are missing data in the pooling subregion near the main diagonal, the missing value is calculated according to 0.

The fully connected layer in the convolutional neural network is the same as the implicit layer in the traditional feedforward neural network. The input of the fully connected layer is one-dimensional data, there is no spatial structure feature, and no feature can be extracted. After the feature extraction in the convolutional and pooling layers, a nonlinear combination is performed in the fully connected layer to achieve classification or regression.

### 3.2. Error Backpropagation Process

The error backpropagation part of the fully connected layer is the same as the traditional feedforward neural network’s. The pooling layer does not contain any parameters or learning process, so there is no need for parameter derivation and gradient descent update in the pooling layer, and the backpropagation in the convolutional layer needs to take into account the size adjustment [22]. Backpropagation is performed after forward propagation to correct the convolutional kernel and fully connected layer parameters of the CNN model until the specified number of iterations is reached.

## 4. Simulation and Result Analysis

In the simulation experiments, an ideal ULA with eight array elements is selected, and the element spacing is λ/2, where λ denotes the wavelength. The target airspace range is [−60°,  60°]. Three narrow-band incoherent signals arrive at the line array from different azimuthal angles. The DCNN structure is the same as that described in Section 3.1 above, the difference is that the input data is the upper triangular array, the number of convolutional kernels in each layer is 12, 12, and 6, the size of the convolutional kernel is 3×3, the activation function is the Leaky ReLU function with the slope of the negative region a=0.1, the pooling layer is selected as the maximum pooling criterion with a size of 2×2, the depth of the fully connected layer is 3, and the number of neurons in each layer is 1500. The size of the training set is 50,000 and the size of the test set is 100. However, the angle information of the input data of the test set follows the actual angle of incidence rather than the discrete angle.

Usually, the root-mean-square error (RMSE) is used to measure the DOA estimation performance, which is defined as:

(10) $$\mathrm{RMSE}=\sqrt{\frac{1}{MQ}\sum_{i=1}^{Q}\sum_{j=1}^{M}{\left({\tilde{\theta }}_{ij}^{\prime }-{\tilde{\theta }}_{ij}\right)}^{2}},$$

where Q is the test set capacity, M is the number of signal sources, and ${\tilde{\theta }}_{ij}^{\prime }$ and ${\tilde{\theta }}_{ij}$ are the DOA estimation and true value of the j-th source in the i-th test, respectively. In this paper, assuming that the incident angle is 25.3° and the step size is 1, the ideal output is 25° according to the assumptions of the model. The difference between the actual output (which may be 24°, 25°, or 26°) and the ideal output (25°) is used as the basis for calculating the RMSE.

### 4.1. Determination of Discrete Angle Interval

**Figure 5:**
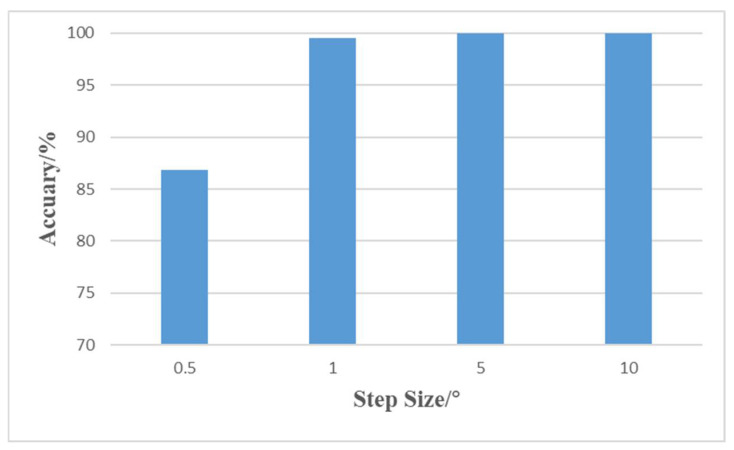
Accuracy of model output for different step sizes.

In determining the discrete angle interval, the effect of step size on the resolution of DOA estimation, accuracy, and the time-space cost of model training is considered. In this paper, 0.5, 1, 5, and 10 are used as discrete angle intervals (i.e., step size) for training, respectively. The step size is the resolution size, and the smaller the step size, the higher the resolution; in terms of accuracy, the model accuracy increases with the step size, as shown in Figure 5 below.

Accuracy means the rate of assigning the angle of arrival to the correct angle bin. The accuracy of the experimental output results for all four step sizes reaches more than 85%, among which the accuracy is nearly 100% when the step size is 1, 5, and 10, but when the step size is 0.5, in the output results, the corresponding values of one or several adjacent sequential numbers are 1, except for the corresponding value of the angular sequential number of the signal’s arrival, which affects the final judgment of the DOA. In terms of time/space complexity, as the target airspace is in [−60°, 60°], the output is 1 × 241 when the step size is 0.5, 1 × 121 when the step size is 1, 1 × 25 when the step size is 5, and 1 × 13 when the step size is 10. The output of the model is a sequence of zeros and ones, and the length of this sequence is influenced by the step size. As the step size decreases, the number of output elements increases, and the higher the number of output elements, the higher the time and space complexity, and the more time and memory are required for model training, the training time and memory requirements increase exponentially. In addition, the signal model and covariance matrix are more similar to the actual received data when the step size is 0.5 and 1 compared to 5 and 10. 

By fully considering the effects of computing efficiency, accuracy, and resolution, in this paper, the step size is chosen to be 1. Although this leads to a loss of resolution accuracy compared to the actual angle of incidence or smaller discrete angular intervals, this loss of accuracy is acceptable in most cases.

### 4.2. Effect of the Number of Snapshots on the Performance of DOA Estimation

**Figure 6:**
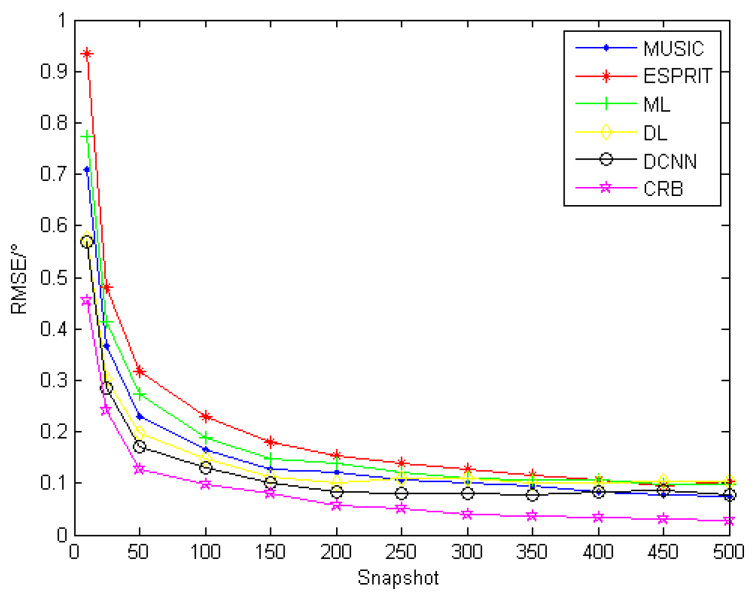
RMSE of each algorithm with different numbers of snapshots.

The snapshot number is the number of sampling points of each array element in the time domain, and usually, the snapshot number will have some influence on the DOA estimation performance. In this set of simulation experiments, keeping the other simulation parameters constant and the signal-to-noise ratio (SNR) as 10 dB, the array-received data with snap counts of 10, 25, 50, 100, 150, 200, 250, 300, 350, 400, 450, and 500 are designed for simulation experiments, and the MUSIC algorithm, ESPRIT algorithm, maximum likelihood, Deep Learning (DL) algorithm, and DCNN method proposed in this paper are used to perform DOA estimation and calculate the RMSE values. In addition, the Cramer–Rao bound is calculated for the same conditions. Among them, the DL algorithm [20] takes the deep fully connected neural network as an example, and the same discretization method is used for the simulation experiments. In the data input stage, the covariance matrix is transformed into a one-dimensional matrix for the input and completes the training. The simulation results are obtained as shown in Figure 6.

From the above figure, it can be seen that the RMSE of the DOA estimation of all five methods tends to decrease with the number of snapshots; when snapshots increase to a certain level (around 250 in Figure 6), the decreasing trend of the RMSE is not obvious, and in practice, very large numbers of snapshots are often difficult to obtain. The DCNN algorithm proposed in this paper is closer to the Cramer–Rao bound.

### 4.3. Effect of SNR on DOA Estimation Performance

**Figure 7:**
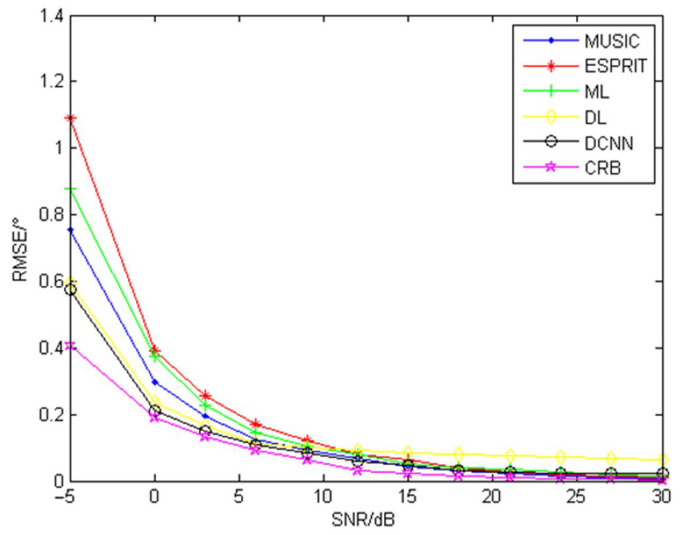
RMSE of each algorithm with different numbers of SNR.

The SNR is the ratio of the power of the output signal to the power of the noise output at the same time, often expressed in decibels. The higher the signal-to-noise ratio, the less noise is generated. In this group of experiments, keeping other parameters unchanged, the snapshot number of 400 is used, and SNRs of −5 dB, 0 dB, 3 dB, 6 dB, 9 dB, 12 dB, 15 dB, 18 dB, 21 dB, 24 dB, 27 dB, and 30 dB are used for simulation experiments. Comparing the RMSE of DOA estimation by the MUSIC algorithm, ESPRIT algorithm, maximum likelihood, the Deep Learning algorithm DCNN method proposed in this paper, and the Cramer–Rao bound (CRB), the simulation results are obtained as shown in Figure 7.

From the above figure, it can be seen that changes in the SNR have significant influence on the DOA estimation accuracy of all five algorithms; the higher the SNR, the lower the RMSE, and the RMSE decreases less and less as the SNR increases continuously; when the SNR is less than 12 dB, the RMSE of the DOA estimation of all five algorithms is significantly affected by the SNR, and the RMSE decreases significantly with the SNR; as the SNR increases further, the RMSE decreases slowly with the further increase in SNR. Among the five algorithms, the DL and the DCNN algorithm proposed in this paper perform well at low SNRs, but when the SNR is greater than 12 dB, the DL algorithm does not perform as well as the other four methods. The DCNN algorithm is significantly better than the other algorithms when the SNR is less than 15 dB at a snapshot number of 400, the performance of MUSIC, ESPRIT, ML and DCNN algorithms is nearly the same when the SNR exceeds 15 dB, and the DCNN is closest to the Cramer–Rao bound.

### 4.4. Effect of Upper Triangular Array as Input on the Efficiency of Operation

As the array output covariance matrix is a matrix symmetric about the main diagonal, the upper triangular array of the covariance matrix is chosen as the input of the DCNN model in this paper. Taking the number of array elements as 6, 8, 10, and 12, the structure of the deep convolutional neural network is kept unchanged, and the upper triangular array and the square array are used as the input, with SNR = 10 dB and snapshot number = 400. Triu represents the upper triangular matrix, Matrix represents the square matrix, and the mean square error values of DOA estimation are shown in Table 1:

As can be seen from the above table, model training using the upper triangular and square arrays produces similar results with no significant difference in the estimated performance. As the network structure is the same when the upper triangular array and the square array are used for model training, respectively, there is almost no effect on the design of the convolutional kernel in the convolutional layer, but there is an effect on the number of convolutional operations and the memory size for storing the convolutional results. Taking the number of array elements as 6, 8, 10, and 12, the structure of the deep convolutional neural network remains the same, and the upper triangular array and the square array are used as inputs. The number of convolutional operations or maximum pooling operations generated by the two input patterns in the convolutional and pooling layers is shown in the following table:

In Table 2, C1, C2, and C3 denote the first, second, and third convolutional layers, respectively, P1 denotes the pooling layer, and SUM denotes the sum of the number of convolutional and pooling operations performed. It can be seen from the table that as the number of array elements increases, the number of operations increases, and both time complexity and space complexity increase. With the same number of array elements, the number of operations is greatly reduced by using the upper triangular array as the model input compared with the square array, and the higher the number of array elements, the higher the proportion of operations reduced by using the upper triangular array, as well as the proportion of total operations reduced by convolution pooling when the number of array elements is 6, 8, 10, and 12. It can be seen that the time complexity and space complexity can be greatly reduced by using the upper triangular array as an input in the convolution and pooling layers.

**Figure 8:**
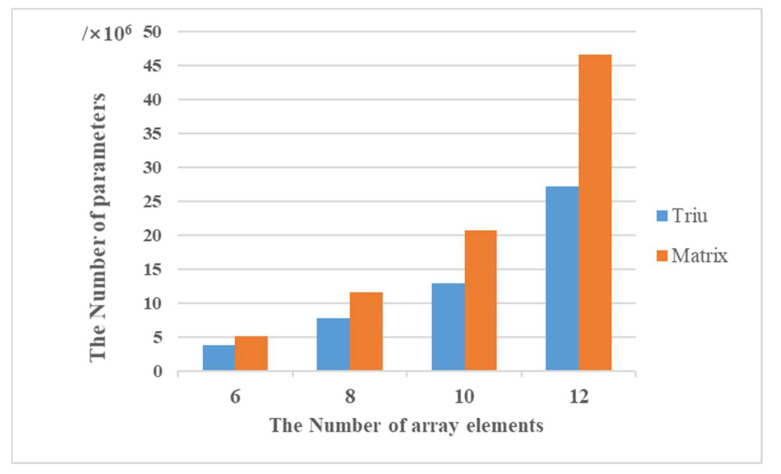
Comparisons of the number of intermediate parameters required under different inputs.

Affected by the structure of the full connection layer, in the whole training process of the DCNN model, the full connection layer has the greatest impact on the time complexity and space complexity. In the case of the same number of hidden layers and the number of neurons in each layer, the larger the input scale is, the higher the computational complexity rate is. After the convolution layer and the pooling layer, the number and size of the feature map generated by the two input modes are still the same, but the feature map generated by the input mode of the upper triangular matrix is still the upper triangular matrix, and the feature map generated by the input mode of the square matrix is still the square matrix, i.e., the size of the feature map generated by the two inputs is N×N, but the former is the upper triangular matrix and the number of elements is $\left(N^{2}+N\right)/2$, but the latter contains the number of elements as $N^{2}$. Therefore, when the feature map is expanded in the full connection layer, the number of intermediate parameters required is also very different, as shown in Figure 8.

The unfolding process of the fully connected layer is equivalent to a convolution process, where the size of the convolution kernel is equal to that of the feature map, and the number of convolution kernels is equal to the number of neurons in the first hidden layer. Although the neural network structures in these two kinds of input modes are identical, the intermediate variables required in the unfolding process are not the same, due to the different number of elements contained in the two convolution kernels when the feature map in the two input modes is convolved. From the Figure 8, it can be seen that as the number of array elements increases, the intermediate variables required in the unfolding process of the upper triangular array and the square array increase, the gap between them further increases, and the reduction in intermediate variables can speed up the operation process and save operation memory. Therefore, it can be seen from both the Figure 8 and the Table 2 that the advantage of using the upper triangular array (or lower triangular array) as an input compared to the square array is that it can fully extract the matrix features and ensure the DOA estimation performance, while effectively reducing the time complexity and space complexity of the deep convolutional neural network in the training process, thus improving the training efficiency.

## 5. Discussion

With the continuous development of deep learning and the increasing complexity of the actual electromagnetic environment, the research of the DOA estimation method based on deep learning provides a new solution idea for this kind of problem. On this basis, this paper proposes a DOA estimation algorithm based on the DCNN, which takes the upper triangular array of the covariance matrix of the discrete received data as the data input, and the output is a spatial angle sequence. The angles corresponding to the first several maxima in the output sequence are the estimated angles under the premise of the known number of sources, so as to achieve DOA estimation of multiple sources. The simulation experiments show that the algorithm outperforms the MUSIC algorithm, ESPRIT algorithm, ML algorithm, and fully connected deep learning algorithm in the case of low SNR and small snapshots, and the algorithm performance is almost the same as those of the MUSIC algorithm, ESPRIT algorithm, and ML algorithm with high estimation accuracy in the case of high SNR and large snapshot. The DCNN is closer to the Cramer–Rao bound under all conditions; meanwhile, compared with the square matrix as an input with the DCNN model, the upper triangular array as an input can effectively reduce the time and space complexity of the model training process and greatly improve the computing efficiency.

## Figures and Tables

**Table 1:** RMSE comparison of Triu and Matrix arrays as inputs with different numbers of array elements.

Array Elements	6	8	10	12
Triu matrix	0.0873	0.0864	0.0852	0.0835
Matrix	0.0882	0.0857	0.0844	0.0837

**Table 2:** Comparisons of the number of operations between Triu and Matrix arrays as inputs in different layers with different numbers of array elements.

Number		C1	C2	C3	P1	SUM
6	Triu	432	3024	8640	2592	14,688
	Matrix	768	5184	13,824	3456	23,232
8	Triu	660	5184	18,144	5184	29,172
	Matrix	1200	9216	31,104	7776	49,296
10	Triu	936	7920	31,104	8640	48,600
	Matrix	1728	14,400	55,296	13,824	85,248
12	Triu	1260	11,232	47,520	18,144	78,156
	Matrix	2376	20,736	86,400	31,104	140,616

## Data Availability

The data presented in this study are available on request from the corresponding author. The data are not publicly available, due to the data in this paper not being from publicly available datasets but obtained from the simulation of the signal models listed in the article.
